# Investigating changes in care patterns and lessons learned during COVID-19 pandemic – an exploratory, convergent mixed method study at two university emergency departments in Germany

**DOI:** 10.1186/s12873-026-01528-5

**Published:** 2026-03-10

**Authors:** Jennifer Hitzek, Bettina Völzer, Martina Schmiedhofer, Dörte Huscher, Anja Alberter, Miriam Mayer, Martin Möckel, Anna Slagman

**Affiliations:** 1 Emergency and Acute Medicine, Campus Mitte and Virchow-Klinikum, Charité – Universitätsmedizin Berlin, Corporate Member of Freie Universität Berlin, Humboldt Universität zu Berlin and Berlin Institute of Health, Charitéplatz 1, 10117 Berlin, Germany; 2 Institute of Clinical Nursing Science, Charité – Universitätsmedizin Berlin, Corporate Member of Freie Universität Berlin, Humboldt Universität zu Berlin and Berlin Institute of Health, Charitéplatz 1, 10117 Berlin, Germany; 3 Institute of Biometry and Clinical Epidemiology, Charité – Universitätsmedizin Berlin, Corporate Member of Freie Universität Berlin, Humboldt Universität zu Berlin and Berlin Institute of Health, Charitéplatz 1, 10117 Berlin, Germany

**Keywords:** COVID-19, Emergency department, Lessons learned, Mixed-method, Nursing, Pandemic preparedness, Patient care

## Abstract

**Background:**

The COVID-19 pandemic posed particular challenges for medical staff in emergency departments (ED). With the end of the pandemic declared by the WHO, the focus shifted to the pandemic preparedness of EDs. The present study aims to identify changes in care patterns in emergency care during the COVID-19 pandemic and derive lessons learned for future health crises.

**Methods:**

An exploratory, convergent mixed method design was conducted. Qualitative data were collected through semi-structured interviews with ED staff. The focus was on their perspectives and perceived experiences of changes in work routines as well as lessons learned during the COVID-19 pandemic. The data were analyzed using thematic analysis. In parallel, routine data from ED patients were descriptively analyzed to examine differences in ED utilization, care pattern and patient flow during the COVID-19 pandemic compared to 2019. Group comparisons and mixed-effects models were used to identify changed patterns and their respective drivers.

**Results:**

Sixteen interviews and 56,842 patient records were analyzed. Overall, a decline in patient numbers was observed during the COVID-19 pandemic as well as changes in length of stay, referrals to ICU and in-hospital mortality compared to 2019. But rapid adjustments required and the accompanying flood of information led to an increased workload. Medical care in the ED was shaped by two overarching dilemmas: (1) Providing optimal care while conserving resources, (2) Delivering optimal treatment in compliance with hygiene guidelines. A shift in self-perception – from helpers to endangerers – had to be newly integrated into role understanding and patient care. To enhance the preparedness of the ED for future pandemics, six key areas have been identified: (1) Avoid Information overload and improve communication, (2) Staff safety and emotional support, (3) Early detection of infections, (4) Hygienic distancing, (5) Resource management in patient care, (6) Efficient discharge management.

**Conclusions:**

To enhance the pandemic preparedness of EDs, an interdisciplinary approach was identified as crucial. This involves a combination of organizational adaptations, effective communication pathways, and emotional support for medical staff for advancing response capacity, resilience, and operational adaptability amidst future pandemics.

**Registration:**

www.drks.de DRKS00023117. Registered 21/09/2020.

**Supplementary Information:**

The online version contains supplementary material available at 10.1186/s12873-026-01528-5.

## Background

“The wish that COVID-19 would just leave us, I don’t think that will happen — but hopefully at some point it’ll be less in-your-face.“, Inteview_1. On 5 May 2023, the World Health Organization (WHO) lifted the status of “Public Health Emergency of International Concern” and thereby declared the end of the acute phase of the COVID-19 pandemic. Consequently, attention has moved towards the lingering effects and lessons learned from the COVID-19 pandemic. It can be assumed that extreme epidemics and pandemics will occur more frequently in the future. The driving factors are: travel, urbanization, climate change, increased human-animal contact and shortage of health workers [1, 2]. In line with the WHO’s emergency response cycle – prevention, preparedness, response and recovery – it is now important to focus on recovery. This stage of the cycle uses the experience gained so far to better prepare emergency departments (EDs) for future pandemic events [3]. An international study found that 97% of the EDs developed and implemented protocols for screening, testing, and treating patients with suspected SARS-CoV-2 infections, however, a third of respondents did not have a pandemic preparedness plan before the COVID-19 pandemic [4]. In Germany, all federal states had a federal pre-existing pandemic preparedness plan, which was available during the pandemic as a guiding framework [5]. Each German state had plans for strengthening and expanding the workforce while 13 of 16 included plans on task-shifting or skill-mix indirectly [5]. According to Maccaro et al., post-pandemic reflection and learning for resilience is one of the most important steps in emerging stronger from the pandemic, pursuing evidence-based policy and preparing the healthcare system for future pandemics [6].

Healthcare staff in EDs were particularly challenged during this time. They had to quickly identify SARS-CoV-2 infections and integrate infection control measures into medical and nursing care, as well as familiarize themselves with the treatment of the new infectious disease [7]. Working on the frontline of the pandemic had multiple impacts on the wellbeing as well as physical and mental health of healthcare workers. Nurses and physicians in EDs were affected significantly by changes in their work routines, alongside the wider stressors and anxieties of living and working through a global pandemic. As a result, working in the ED was described as extremely stressful, morally damaging and exhausting [8, 9]. The main reasons for increased stress were the fear of infecting family members, physical or mental exhaustion and change in tasks [10]. Morawa et al. examined mental distress in healthcare workers in EDs during the COVD-19 pandemic and found a prevalence of 20.9% for anxiety symptoms and 19.1% for depression symptoms [11]. As a lesson learned, the “Simic Support Framework” was developed to provide a guideline for organizations, which can be used to foster holistic wellbeing in the ED [12]. The implementation of a rapid response infrastructure in pandemic events has also been prove to be helpful to reduce the workload in the ED [13].

Despite numerous studies on the impact of the COVID-19 pandemic on EDs, one important question remains unanswered: How have patterns of care in EDs changed and what are the long-term implications for ED preparedness for future pandemics? In Germany in particular, we are not aware of any studies that examine a holistic analysis of the changes in patient care in EDs during the COVID-19 pandemic and their implications for future pandemics. Therefore, the aim of the present study is to identify changes in care patterns in emergency care during the COVID-19 pandemic and to derive lessons learned for future health crises. While this study is grounded in the specific context of the COVID-19 pandemic, the identified changes in ED utilization, care processes and staff experiences address generic vulnerabilities of emergency care systems that are likely to recur in future epidemics and other large-scale health crises. By translating these lessons into specific recommendations for communication, staff protection, infection detection, hygiene concepts and resource management, our findings support long-term strengthening of ED resilience and preparedness beyond the COVID-19 pandemic.

## Methods

This retrospective observational, mixed method study addresses changes of care in EDs during the COVID-19 pandemic, using an exploratory, convergent mixed-method approach. The study was conducted from November 2020 to April 2021 at two EDs of the Charité – Universitätsmedizin Berlin, Germany, a tertiary care university hospital in the inner-city area of Berlin.

**Fig. 1:**
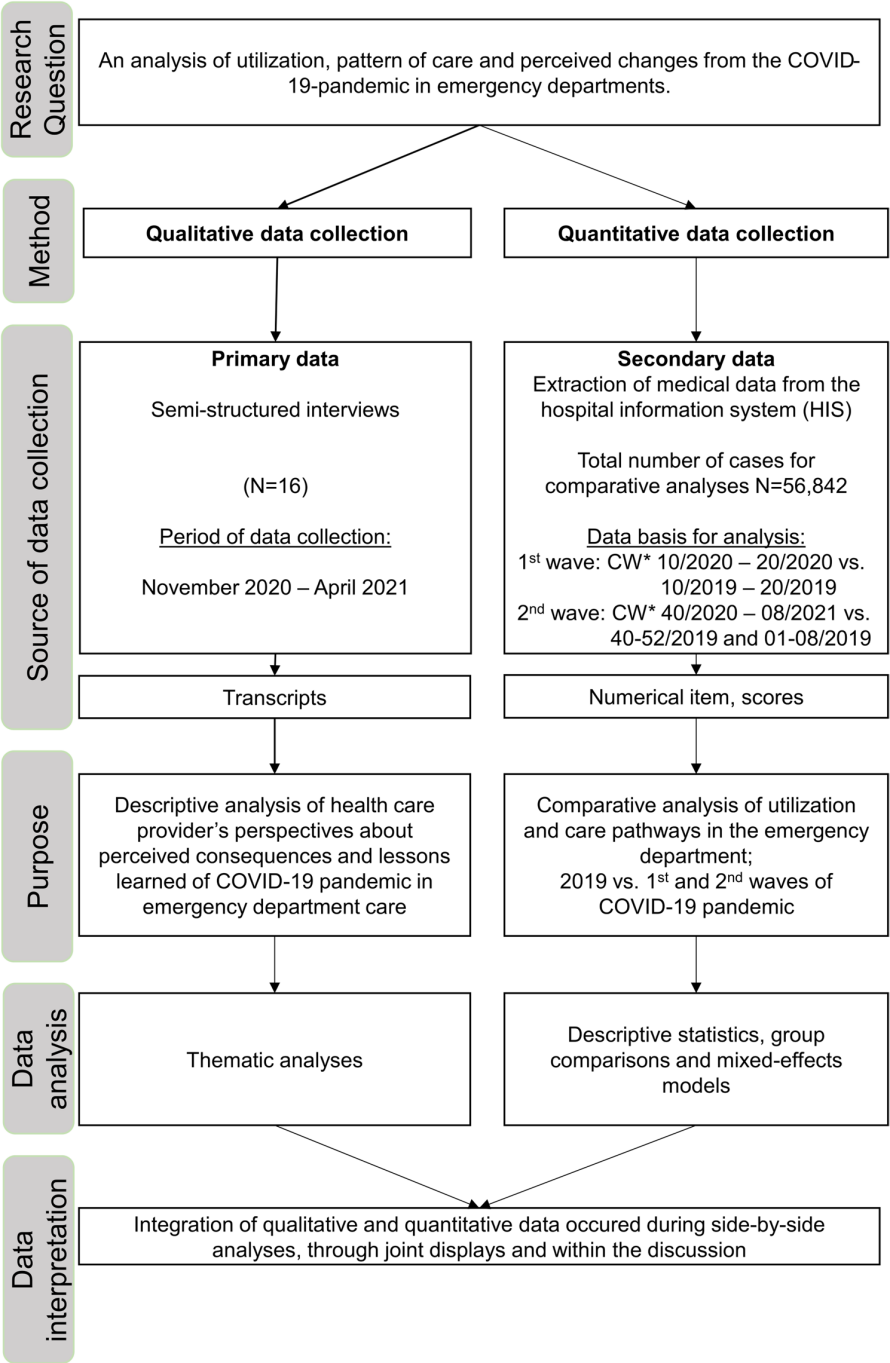
Flowchart of the exploratory, convergent mixed methods design. *CW =calendar weeks

The study employs an exploratory research design aimed at developing a comprehensive understanding of the phenomenon, identifying underlying patterns and relationships, and deriving lessons learned to strengthening the pandemic preparedness of EDs. A convergent mixed method design was conducted; quantitative and qualitative data were collected in parallel, analyzed separately, and then integrated into the subsequent analysis and interpretation [14]. A detailed description of the study design, is shown in Fig. 1. Pragmatism was the underlying research paradigm and basis for the choice of the mixed method design, where research question drives the research methods [15]. The objective reality of ED utilization and changes in care patterns were examined with the experiences and subjective perceptions of day-to-day work in the ED during the COVID-19 pandemic. In this study, care patterns are operationally defined as: (1) quantifiable changes in ED processes and outcomes during the COVID-19 pandemic compared to pre-pandemic (2019) values. These include emergency department utilization (number of patients per calendar week), patient characteristics (age, sex, Charlson Comorbidity Index), treatment urgency (categories of the Manchester Triage System), time metrics (time between admission and triage, length of stay), disposition (inpatient/outpatient, intensive care admissions), and in-hospital mortality. As well as qualitative data (2), perceived changes in patient care and ED organization from the perspective of medical staff.

Lessons learned are defined as higher-level themes derived from the inductive thematic analysis of semi-structured interviews with ED staff, integrated with quantitative analyses of changes in care patterns. These themes capture actionable recommendations for enhancing future pandemic preparedness of EDs.

The combination of both methods will produce a robust data set to address the research question. A mixed method design was chosen to provide deeper insight into the topic by both complementing one set of findings with another and expanding the scope of inquiry to cover different but related aspects, as described by Greene et al. [16]. In addition, the complementary integration strategy of data post-analysis via side-by-side comparison of themes and statistical outcomes can balance the disadvantages of each method and highlight possible convergences, divergences or relationships [16, 17].

### Strategy of sampling and recruitment

At the EDs, participants for semi-structured interviews were recruited through purposeful sampling to capture diverse perspectives on pandemic-related changes of care in the ED [18]. The study aimed to interview a broad range of medical staff, including physicians and nurses involved in patient care. In addition to professional affiliation, participants’ age and their experience in the ED were taken into account during the recruitment process. Furthermore, an effort was made to purposively include individuals in managerial or leadership roles in order to obtain a comprehensive perspective on ongoing transformations within ED. Recruitment occurred via two main strategies: the primary researcher introduced the study during morning team timeouts, and flyers and posters were distributed in breakrooms. Additionally, five participants were recruited through intermediaries during the study period.

### Data collection

#### Qualitative data

The semi-structured interview guide was designed according to the guidelines of Helfferich et al. [19] and previously discussed and piloted by the research team. To start the interview, we asked: “How did you personally experience the daily work routine in the ED during the COVID-19 pandemic and what changes did you experience?”. The order of questions depended on the course of the conversation. Study participants could choose between face-to-face interviews or an online meeting. Audio recordings were used for documenting face-to-face interviews and video recordings were used for online meetings respectively. Interviews were conducted in German, transcribed according to the rules of Kuckartz and anonymized [20].

The interview was conducted during working time and participants received a study information and provided written informed consent.

#### Quantitative data

Secondary data of ED visits were extracted electronically from the hospital information system (HIS) and anonymized. The dataset included information about medical treatment and care pathways during the ED visit, and, if applicable, information about the subsequent inpatient hospital treatment of the year 2019 and the first and second wave of the COVID-19 pandemic in Germany at one ED (CVK); age, sex, time of admission, time of discharge, Manchester triage, status of SARS-CoV-2 infection, admissions to intensive care unit, in-hospital mortality. The year 2019 was used as the reference frame and compared with the first and second wave of the COVID-19 pandemic to examine changes in ED utilization and patterns of care. Waves of the COVID-19 pandemic were defined in accordance with Schilling et al. [21] using calendar weeks as unit of analyses; first wave was defined from calendar week 10/2020 to 20/2020 and second wave from calendar week 40/2020 to 8/2021. All patients who visited the ED during the study periods and were older than 17 years were included. Patients in the pediatric ED and patients who visited the ED due to an accident at work were excluded.

### Data analyses

#### Qualitative data

Semi-structured interviews were analyzed using thematic analyses according to Braun et al. [22].

Thematic analyses were conducted using a realistic approach, assuming a unidirectional relationship between experiences and meanings [23]. The process began with a case-based summary of each interview. Six interviews were independently coded by two colleagues, identifying initial codes and themes inductively. A purely inductive analytical approach was adopted in order to preserve the exploratory nature of the study and to enable perspectives and phenomena to be captured in their entirety without being constrained by predetermined topics. Codes and themes were discussed for consistency and clarity to ensure reliability and topic diversity [22, 24]. Two researchers used this preliminary coding frame for the independent analysis of remaining interviews, supplemented by additional inductive codes. Through iterative team discussions and triangulation, codes were condensed into higher‑level themes to ensured analytic rigor and captured staff experiences across pandemic waves. Analyses were conducted using MAXQDA 2022, Berlin.

#### Quantitative data

Changes in utilization and patterns of care in the ED were examined using descriptive analysis. Metric variables are presented using median (Mdn) and interquartile range (IQR). Categorial variables are presented using absolute and relative frequencies. A p-value of *p* ≤ 0.05 was considered as significant.

To obtain a general overview, sex and age differences in ED utilization were analyzed using Wilcoxon rank-sum test, comparing 2019 and the first and second wave of COVID-19 pandemic. The descriptive analyses of these variables were carried out using aggregated data at the calendar week level. Due to the different number of weeks in 2019, 2020, and 2021, it was necessary to adjust the number of weeks for pairwise statistical analysis; all three years were standardized to 52 calendar weeks, so that holidays, such as Christmas and New Year’s Eve, fell into the same calendar weeks. Subsequently, the analyses were adjusted for seasonal fluctuations in the utilization of EDs. The pairwise comparison between 2019 and the two waves at the calendar week level were performed using the Wilcoxon signed-rank test. A linear mixed model was performed to analyze differences in utilization of the ED (proportion of a calendar day in the respective calendar week) comparing the numbers of the first and the second COVID wave with the pre-pandemic year 2019, with fixed effects for the wave, calendar day and interaction of both and random effects for the intercept.

Charlson Comorbidity Index (CCI) was calculated, using the method of Quan et al. [25, 26] to describe patients more precisely in terms of their comorbidities and mortality risk. The CCI was categorized according to Charlson et al. 0 points, 1–2 points, 3–4 points and ≥ 5 points: Higher scores are associated with more comorbidities, increased one-year mortality risk and resource consumption [27, 28]. CCI categories were then compared between 2019 and both waves of COVID-19 pandemic using Chi-square test.

Group comparisons for the following categorial variables were performed with the Chi-square test: infection status (patients with and without SARS-CoV-2 infection), treatment urgency by Manchester triage (“urgent” (MTS 1–3) and “non-urgent” (MTS 4 and 5) [29]), case type (inpatient, outpatient), admission to intensive care unit and in-hospital mortality.

Differences in time from admission to triage and length of stay in the ED between the first and second wave were analyzed using Kruskal-Wallis Test. Analyzing the differences in time from admission to triage and length of stay in the ED between patients with and without SARS-CoV-2 infection during the first and second wave, the Wilcoxon rank-sum test was used.

All statistical analyses were performed by using IBM SPSS Statistics 30, New York.

#### Ethics and registration

The research project was approved by the ethics committee of Charité –Universitätsmedizin Berlin, Germany (EA1/163/20), and the institutional data protection department. Neither patients nor the public were involved in the design, conduct, or dissemination plans of this research. The study was registered at www.drks.de (DRKS00023117) on 21/09/2020.

## Results

Sixteen interviews of ED staff – eight online and eight face to face – were included in this analysis. For the quantitative analyses this study included 56,842 ED patients. Patients were in median 46 [IQR 31;65] years old and the sample was evenly distributed in regard of sex. A confirmed SARS-CoV-2 infection was found in 1,113 (2.0%) of these patients. Characteristics of both samples are provided in Table 1.

**Table 1:** Descriptive characteristics of study population

Study Characteristics Qualitative data – ED* staff ( ***N*** ** = 16)**	
Age in years Md, [IQR**]	36 [31;43]
Sex (male) in %, (n)	43.8 (7)
Duration of interview in min. Md, [IQR]	43.0 [31.5;50.5]
Occupation in %, (n)	Nurse 56.3 (9), Physician 43.7 (7)
Management position in %, (n)	18.8 (3)
Work experience in ED in years Md, [IQR]	10.5 [5.8;12.5]
**Study Characteristics Quantitative data – ED patient records** **(*N* = 56,842)**	
Age in years Md, [IQR]	46 [31;65]
Sex (male) in %, (n)	49.9 (28,345)
Proportion of patients with positive SARS-CoV-2 infection in % (n)	2.0 (1,113)
**Classification of treatment urgency** Proportion of less urgent treatment (MTS*** 4–5) in % (n) Proportion of urgent treatment cases (MTS 1–3) in % (n)	39.7 (21,886) 60.3 (33,187)
**Length of stay in the ED in hours Mdn [IQR]**	18.7 [8.3;46.9]
**Case type** Inpatient treatment in %, (n) Outpatient treatment in %, (n)	28.5 (16,205) 71.5 (40,673)
Admission to ICU**** in % (n)	5.8 (3,276)
In-hospital mortality in %, (n)	1.4 (775)

*Emergency department, **Interquartile range, ***Manchester Triage System, ****Intensive Care Unit

### Changes in patient profile and case-mix across pandemic waves

During the first and second wave of the COVID-19 pandemic, weekly ED visits decreased by 33% and 25% respectively compared to 2019. The median number of ED patients per week decreased from 990 (IQR 953–1.036) to 666 (IQR 640–697) in the first wave and 742 (IQR 684–805) in the second wave. Despite fewer patients, staff reported routine work as stressful due to concerns about those staying away out of fear and challenges in managing new patient pathways (Table 2).

**Table 2:** Changes in care pattern of EDs patients (*n*=56,842) of the ED of Charité – Universitätsmedizin Berlin (CVK). Details about utilization, demographics, comorbidity, treatment urgency, care patterns and in-hospital mortality were shown for the pre-pandemic time 2019 as well as the first (10-20/2020) and second wave (40/2020-08/2021) of the COVID-19 pandemic in Germany

		2019	First wave	Second wave	*p*- value	Results from Interviews
Sex (male) in % (*n*)		49.1 (16,229)	51.6 (3,845)	50.6 (8,271)	*p*=0.001	
Age in years, Mdn [IQR*]		45.0 [30;64]	45.0 [31;64]	48.0 [32;67]	*p*<0.001	
**Changes in care patterns**						
Number of ED** patients per calendar week, Mdn [IQR]		990 [953;1,036]	666 [640;697]	742 [684;805]	*p*<0.001	“It wasn’t necessarily just more relaxed work because there were fewer patients. Rather, when the patients came in, you just didn’t know how you were supposed to behave correctly.“, Interview_4.
Charlson Comorbidity Index*** in % (n)	Score 0	50.9 (16,808)	49.7 (3,705)	47.0 (7,684)	*p*<0.001	“Second wave was the complete opposite. We had our usual patient population again. Which is a good thing, because it means that people finally felt confident enough to come to the hospital again. And there was a significantly higher number of COVID patients — many more and much sicker.”, Interview_5.
	Score 1-2	23.4 (7,715)	24.0 (1,789)	23.4 (3,824)		
	Score 3-4	15.9 (5,260)	15.3 (1,142)	17.3 (2,836)		
	Score ≥5	9.8 (3,251)	10.9 (813)	12.3 (2,015)		
Proportion of patients with positive SARS-CoV-2 infection in % (n)		0 (0)	5.5 (408)	4.3 (705)	*p*<0.001	
Proportion of less urgent treatment (MTS**** 4-5) in % (n)		40.1 (12,920)	40.0 (2,908)	38.9 (6,058)	*p*=0.034	
Proportion of urgent treatment cases (MTS 1-3) in % (n)		59.9 (19,296)	60.0 (4,367)	61.1 (9,524)		
Inpatient treatment in % (n)		27.1 (8,939)	28.9 (2,154)	31.2 (5,112)	*p*<0.001	
Outpatient treatment in % (n)		72.9 (24,095)	71.1 (5,295)	68.8 (11,247)		
Time from admission to triage in min. Mdn [IQR]		8.0 [2.0;18.0]	8.0 [2.0; 17.0]	10.0 [4.0; 19.0]	*p*<0.001	“And so before triage, before they even come through the door, there’s basically a mini-triage: temperature’s taken, mask goes on, then you ask …about their main symptoms. That way we’ve limited the surprises.“, Interview_6.
Length of stay in the ED in hours Mdn [IQR]		19.1 [8.4; 47.3]	22.7 [9.1; 47.3]	15.7 [7.9;45.8]	*p*<0.001	“The outflow was even harder. Of course, the patients piled up, because the wards said they wouldn’t take anyone who wasn’t proven negative. But with PCR that only came after 12 hours, and then nobody took the patients off us anymore.“, Interview_7.
Admission to ICU***** in % (n)		4.9 (1,621)	6.7 (500)	7.1 (1,155)	*p*<0.001	
In-hospital mortality % (n)		4.0 (354)	5.1 (110)	5.1 (263)	*p*=0.002	

* Interquartile range, ** Emergency department, *** The Charlson-Comorbidity Score is associated with different one-year mortality risk: Score 0 =12%, Score 1-2=26%; Score 3-4=52%, Score ≥5=85%. **** Manchester Triage System, ***** Intensive Care Unit

The mixed-effects model examined variations in ED utilization across weekdays and their interactions with distinct COVID-19 pandemic waves, using Monday and the year 2019 as reference points.

**Fig. 2:**
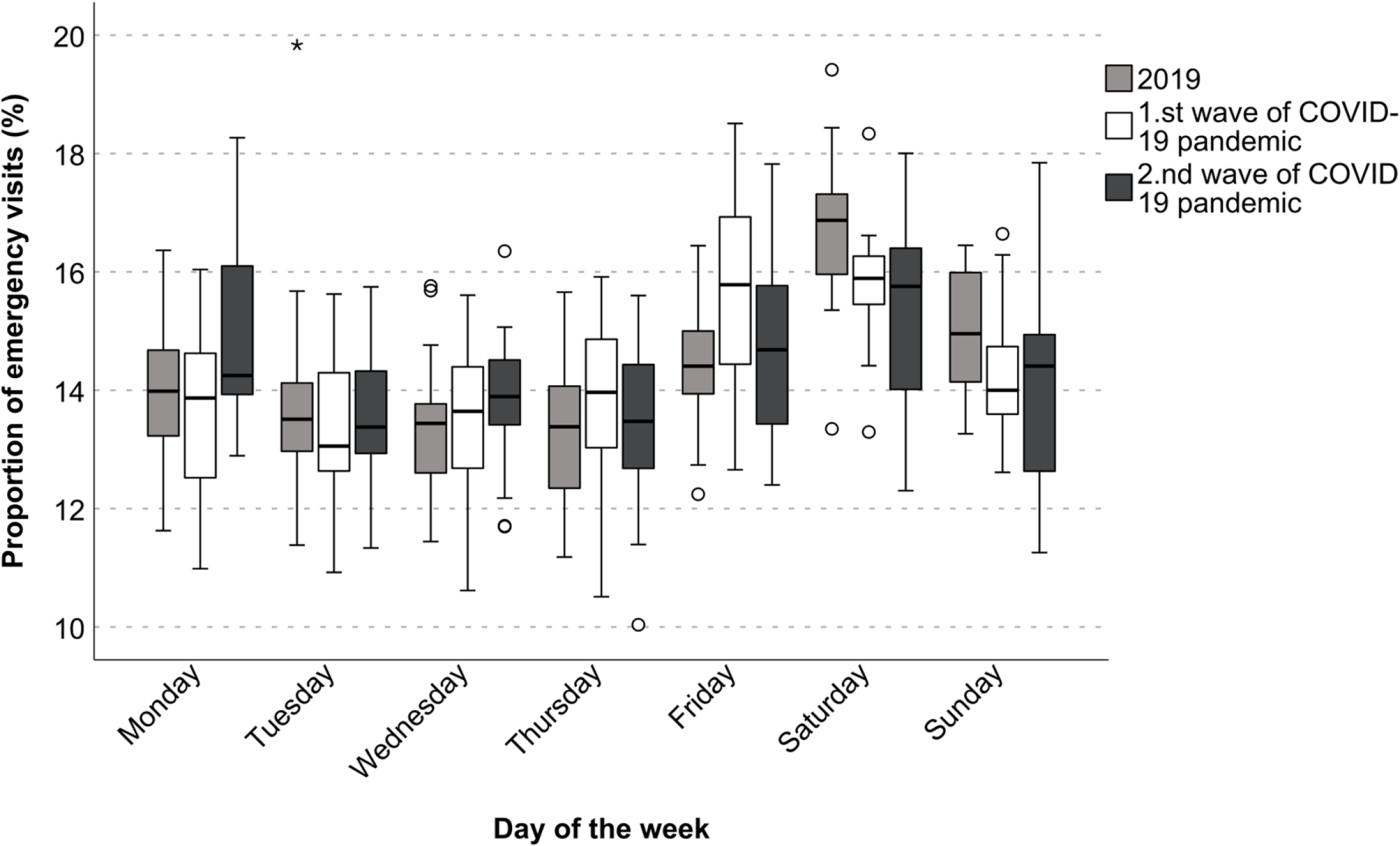
Comparison of the weekly ED utilization distribution between the pre-pandemic period (2019), the first (10–20/2020) and the second wave (40/2020-08/2021) of the COVID-19 pandemic in the ED of Charité – Universitätsmedizin Berlin (CVK)

Weekday-specific interactions with the wave revealed an elevated utilization of EDs on Fridays during the first wave (1.56; *p* = 0.016), and declined utilization on Tuesdays, Saturdays, and Sundays during the second wave (-1.20, -2.34, -1.86; all *p* < 0.05), see Figure 2.

Patients in the second wave were on median three years older than pre-pandemic and patients in the first wave of the COVID-19 pandemic. Significant differences in infection status were observed between the waves: While SARS-CoV-2 positive patients were on median 7 years younger during the first wave, they were, in contrast, 10 years older during the second wave compared to the pre-pandemic period.

Charlson Comorbidity Index scores shifted towards higher comorbidity, especially among SARS-CoV-2 positive patients. An overall rise in treatment urgency was observed. In the first wave, urgent cases were markedly less frequent among SARS-CoV-2 positive patients (–19.4%) relative to negatives, whereas in the second wave this pattern reversed (+ 8.2%).

The share of inpatient cases increased from 27.1% pre-pandemic to 31.2% during the pandemic. Among SARS-CoV-2 positive patients, inpatient admissions were reduced in the first wave (–15.8%) but nearly doubled compared with negatives in the second (29.8% vs. 62.2%).

ICU admission and in-hospital mortality increased overall, with a 3.4-fold higher ICU admission rate for patients with SARS-CoV-2 infection in the second wave (6.4% vs. 22.0%) and a 2.5-fold higher in-hospital mortality among patients with a SARS-CoV-2 infection in both waves. Staff perceived these changes as a shift from younger, less comorbid patients in the first wave to “many more and much sicker’ patients” in the second wave (Table 2).

Statistical results on differences in the ED care processes over the course of the COVID-19 pandemic can be found in Table 2. Differences based on infection status within the waves of the COVID-19 pandemic can be found in Table 3.

**Table 3:** Comparisons of care patterns according to the status of SARS-CoV-2 infection between the pre-pandemic period 2019 as well as the first (10–20/2020) and second wave (40/2020-08/2021) of the COVID − 19 pandemic, of patients in the ED of the Charité – Universitätsmedizin Berlin (CVK)

		Wave of the COVID-19 pandemic	Infection status		p- value
			Negative	Positive	
Age in years Mdn [IQR*]		First	46.0 [31.0; 64.0]	39.0 [29.0; 55.0]	*p* < 0.001
		Second	47.0 [31.0;67.0]	57.0 [40.0;71.0]	*p* < 0.001
Sex (male) in % (n)		First	51.9 (3,653)	47.1 (192)	*p* = 0.058
		Second	50.2 (7,856)	58.9 (415)	*p* < 0.001
Treatment urgency in % (n)	Less urgent treatment cases (MTS** 4–5)	First	38.9 (2,684)	58.3 (224)	*p* < 0.001
		Second	39.2 (5,840)	31.0 (218)	*p* < 0.001
	Urgent treatment cases (MTS 1–3)	First	61.1 (4,207)	41.7 (160)	*p* < 0.001
		Second	60.8 (9,039)	69.0 (485)	*p* < 0.001
Charlson Comorbidity Index in % (n)	Score 0	First	49.0 (3,449)	62.7 (256)	*p* < 0.001
		Second	47.6 (7,455)	32.5 (229)	*p* < 0.001
	Score 1–2	First	24.1 (1,694)	23.3 (95)	*p* < 0.001
		Second	23.2 (3,639)	26.2 (185)	*p* < 0.001
	Score 3–4	First	15.7 (1,108)	8.3 (34)	*p* < 0.001
		Second	17.2 (2,695)	20.0 (141)	*p* < 0.001
	Score ≥ 5	First	11.2 (790)	5.6 (23)	*p* < 0.001
		Second	11.9 (1,865)	21.3 (150)	*p* < 0.001
Case type in % (n)	Inpatient treatment	First	29.8 (2,097)	14.0 (57)	*p* < 0.001
		Second	29.8 (4,671)	62.2 (441)	*p* < 0.001
	Outpatient treatment	First	70.2 (4,944)	86.0 (351)	*p* < 0.001
		Second	70.2 (10,983)	37.4 (264)	*p* < 0.001
Time from admission to triage in min. Mdn [IQR]		First	8.0 [2.0;17.0]	9.0 [2.0;26.0]	*p* < 0.001
		Second	10.0 [4.0;18.0]	22.0 [8.0;32.0]	*p* < 0.001
Length of stay in the ED in hours Mdn [IQR]		First	22.7 [9.0;47.4]	24.3 [8.6;46.3]	*p* = 0.830
		Second	16.0 [7.9;46.3]	13.0 [8.0;28.3]	*p* = 0.093
Admission to ICU*** in % (n)		First	6.8 (480)	4.9 (20)	*p* = 0.133
		Second	6.4 (1,000)	22.0 (155)	*p* < 0.001
In-hospital mortality in % (n)		First	4.9 (103)	12.3 (7)	*p* = 0.013
		Second	4.5 (208)	12.5 (55)	*p* < 0.001

*Interquartile range, ** Manchester Triage System, *** Intensive Care Units

### Organizational tension between infection control, patient flow and crowding

ED operations during the pandemic were characterized by persistent tension between infection control, patient flow, and crowding. These competing priorities shaped clinical workflows, resource allocation, and interprofessional dynamics.

Admission-to-triage times remained similar to pre-pandemic levels but increased in the second wave, particularly for SARS-CoV-2 positive patients whose admission-to-triage time was in median 12 min longer compared to negative patients. A screening process was implemented prior to standardized Manchester Triage System to facilitate early detection of infectious patients. Subsequent triage and disposition, primarily nursing tasks, required extensive interprofessional collaboration and communication (see Table 2).

ED length of stay (LOS) rose from 19.1 h pre-pandemic to 22.7 h in the first wave, then declined to 15.7 h in the second wave, below pre-pandemic levels. Staff attributed these dynamics to additional hygienic measures, isolation requirements and limited capacities, which complicated patient placement and discharge and intensified crowding.

“And you always have to bear in mind that every time this room is used, it’s not just the time the patient is in there until they’re moved on. You then have to get a specialist cleaner who does the special clean. So it’s not like usual, where you just wipe it over once and you’re done — you have to get someone special to come and clean it all. And that takes up a lot of time.“, Interview_2.

They described a “vicious circle of crowding” in which waiting for test results, restricted external transfers and limited isolation rooms led to crowding in the ED, increasing basic nursing demands while time and resources remained scarce. Prolonged ED stays increased basic nursing care demands, yet staff perceived patients as underserved, with care reduced to minimum essentials.

“You miss things. When I start my shift and already have twelve patients who’ve been here for 16 hours… They want food. They want to be washed. They want to know what’s going on. They get impatient — after all, they’re in the ED. ‘What’s happening here? Why can’t I go up to the ward?’ All that takes up time, which, honestly, in the ED, you just don’t really have planned for.“, Interview_3.

This created conflict among staff over balancing basic nursing/therapeutic care with acute ED priorities.

Structural adjustments, such as pre-triage screening, real-time PCR for urgent cases, visual floor markings and dedicated coordination roles, were perceived as helpful, but only partially mitigated the tension between infection control standards and patient flow.

“Space is generally always an issue. Hygiene measures and distancing here in the ED as well. At first, we could hardly enforce it, because the sheer mass of patients completely overwhelmed us… how can we increase the safety measures? Then we started marking the floor: ‘Only here may a patient be, only here may a stretcher be.’ And no longer everywhere like before. That worked relatively quickly. We numbered, and it established itself automatically: ‘Patient so-and-so is in Corridor 3.’”, Interview_8.

The interplay of infection control, flow constraints, and crowding thus demanded continuous organizational adaptation throughout the pandemic.

### Transformation of professional roles and identity

Across pandemic waves, staff reported a profound shift in professional self-perception, describing themselves simultaneously as helpers and potential endangerers — constantly aware of being both caregivers and possible source of infection for patients and family members. This dual role extended into their private lives, accompanied by fear of transmitting the virus or being stigmatized.

“If I catch it, it’ll be at work — privately I hardly meet anyone now. I’ve already put up more boundaries in my private life, because there are more infections out there, and I don’t want to make things worse… or, put it this way, I’m afraid people will point the finger at me afterwards just because I work here.“, Interview_11.

The awareness that every patient is now to be seen as a potential COVID case intensified fear and self-protective behaviors.

“Every patient is now to be seen as a potential COVID case, and that really eats away… eats away at my being here, you know. Really, I should be wearing a mask all the time and not touching anyone… Anything with a fever, you still go in at the moment with a second mask. Whether that makes sense I don’t know, but for my head it makes sense. I protect myself twice. I use a visor, I use this face shield, I use this gown and everything else we’ve got and two pairs of gloves. … You’ve… it sounds silly, but you’ve actually become more hygienic, I would say.“, Interview_10.

This fear reshaped professional norms: self-protection became an essential part of patient care.

“That really was something new — that you yourself had to be afraid of catching something, where before it was always just about me not passing it on.“, Interview_12.

Personal protective equipment increased workload and coordination demands, reinforcing the daily conflict between responsibility, safety, and limited resources.

Unexpected positive cases repeatedly triggered fear and self-doubt about exposure and safety.

“…and then there are patients you wouldn’t have suspected. And then the mental wheels start turning — what did I do? What mask was I wearing, how close was I to the patient, did they cough?“, Interview_13.

### Ethical dilemma under constrained conditions

At the pandemic’s peak, rising admissions and ICU rates, prolonged ED stays and limited isolation capacities created recurrent dilemmas in admission and treatment decision. Staff described feeling that they could “only do it wrong.”

“You either discharge someone home from the ED who’s too sick because you want to save bed capacity, or you admit someone who’s too healthy, and then you create problems afterwards because the bed is missing for someone who needs it much, much more urgently,” Interview_14.

This constant weighing between individual and system-level needs caused major stress and emotional strain.

Strict hygiene requirements heavily affected treatment. Measures like limits on non-invasive ventilation and inhalation therapy exhausted staff and created uncertainty and reinforcing the sense of not being able to offer optimal care.

“The additional isolation measures, ultimately also the therapy limitations for COVID patients, that makes it all extremely exhausting … COVID, not COVID, rapid antigen test, positive, negative, PCR…,” Interview_6.

Emergency procedures such as intubations heightened safety concerns and fear of overstepping one’s competence.

“There always has to be a fallback level… it should be ensured for as long as possible that no one has to step in heroically. And I don’t mean heroic in a good way — I mean in an overstrained way, getting into a rescuer position that can really backfire.”, Interview_14.

Treating vulnerable groups like children and dementia patients was particularly difficult due to communication barriers and infection risks.

“Of, course it’s really hard for the patient. If someone comes in with real shortness of breath and we insist they still wear their mask, it’s a challenge for them, but also for us, because obviously it’s a huge risk for us,” Interview_13

Personal protective equipment, while essential for safety, introduced physical and emotional distance, reducing non-verbal communication.

“There’s a lack of humanity somewhere. I see who’s there, I can tell from the smile, the grin, whatever. ‘Oh, you’ve got a great beard,’ or whatever. That’s how you get into a conversation. Now it’s just the eyes. And that distances me from you. I’m not only putting on gloves, I’ve also got a gown, a mask of course, then the face shield, and then people can hardly understand me. You have to talk louder, and that distances me from the patient — I’d say yes, it does,” Interview_10.

Fear of infection limiting time at the bedside and contributing to experiences of superficial psychological support for patients with a positive SARS-CoV-2 status.

Fear of infection further reduced personal contact, leaving many COVID patients feeling isolated.

“With COVID patients themselves, where we knew they were positive, you massively cut down the time you spent with them… because I wanted to minimize exposure time. I do think that COVID patients in our ED were at times quite alone. You’re really isolated — it’s like being a leper, that’s what it’s like,” Interview_5.

Overall, staff described care for COVID patients as emotionally detached and superficial, contradicting their understanding of good care.

### Communication challenges and information overload

During the pandemic, employees faced constant updates due to daily changing hygiene rules, treatment guidelines and a flood of information.

What’s… difficult is when you hear for five days in a row… ‘Well, what we told you yesterday, you can forget that….’… colleagues… they don’t actually want to have so much information., Interview_8.

The constant changes led to uncertainty, confusion, growing stress and, at times, deliberate gradual adaption of update to cope with information overload.

“…that there was a certain… fatigue with the constant new changes. … So… I just skip the change for a week and then I’ll pick it up again two weeks later.”, Interview_9.

Frequent guideline adjustments required continuous communication within interprofessional teams, which became increasingly exhausting over time.

You have to bring everyone along with you in this process, and this rapid distribution of information has been an absolute challenge., Interview_9.

Measures to reduce the number of people in the ED, especially excluding relatives, eased crowding but created new communication burdens.

“We just didn’t have the resources to constantly be the link between the patient and their relatives. That meant, relatives often sat in the waiting area for six or seven hours until someone had time to talk to them. That’s not a nice situation either.“, Interview_8.

### Recommendation for practice and pandemic preparedness

**Fig. 3:**
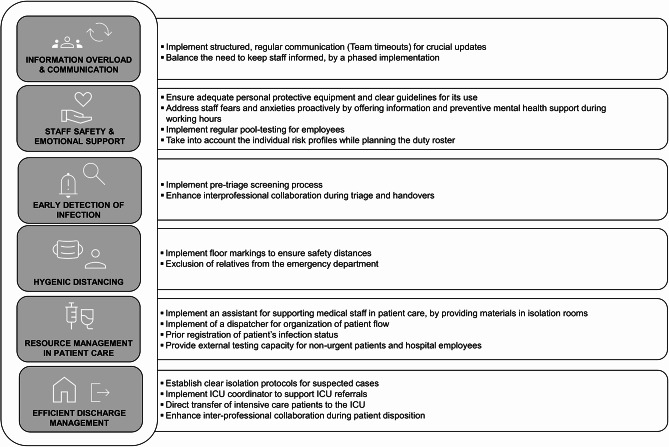
Lessons learned from the EDs during COVID-19 pandemic and recommendations for future pandemic preparedness of ED - perspectives of medical staff 8Interview 1 - 16)

Following convergent parallel design principles, quantitative patterns were analyzed independently of qualitative themes from the thematic analysis and then brought together at the interpretation level to generate conclusions that were incorporated into the recommendations in Fig. 3.

The figure summarizes key areas of action for strengthening the ED in terms of pandemic preparedness, specifically addresses the management of information overload and communication, the safeguarding of staff protection and psychosocial support, as well as the early detection of infections. In addition, measures related to hygienic distancing and the management of scarce resources in both patient care and discharge or transfer processes are outlined.

## Discussion

After overcoming the acute phase of the COVID-19 pandemic, it is now about efficiently and thoroughly processing the experiences and developing action strategies for future pandemics. Particularly ED staff was affected by the COVID-19 pandemic in various ways and can thus provide a valuable contribution to future pandemic preparedness in EDs. The present study identifies changes in care patterns in emergency care during the COVID-19 pandemic and derives recommendations for future health crises. Our study synthesizes quantitative changes in ED care patterns — such as reduced patient visits by 25–33%, increased length of stay, and higher acuity in the second COVID-19 wave — with qualitative insights from 16 staff interviews highlighting dilemmas like resource conservation amid hygiene demands. Our study shows a significant reduction in patient numbers in EDs during the first and second wave of the COVID-19 pandemic, following the general trends in ED utilization in Germany [30, 31] as well as an increased ICU utilization, and an elevated in-hospital mortality rate among COVID-19-positive patients. Suarez and colleagues confirm the results and were able to demonstrate an increased mortality in the ED for patients with SARS-CoV-2 infection in Germany, too [32]. An explanation for the observed increase in critical treatment patterns could be the spread of the more contagious virus variant B1.1.7, which rapidly spread in Germany at the beginning of 2021 [33]. The significant increase in individuals with a higher CCI during the second wave suggests that people with pre-existing conditions were particularly affected by the COVID-19 pandemic [34]. They must be given special consideration when it comes to adapting care structures and preparing EDs for future pandemics. Staff experienced information overload from rapidly changing guidelines, contributing to “guideline fatigue” and heightened stress, which paralleled volatile ED utilization patterns like weekend drops in the second COVID-19 wave. To counteract the feeling of being overwhelmed by constant adjustments and new information in the future, regular team timeouts have proven helpful. During these timeouts, all employees from the ED come together, and current adjustments could be discussed. There was a feeling of being up to date, along with the opportunity to address questions. Employees expressed a desire for this form of interprofessional exchange to be implemented during all shift changes as well. In such acute scenarios, the working hours of medical and nursing staff would then need to be synchronized to ensure ideal information transfer, promote safety in patient care, and reduce stress. These practices align with evidence from EDs during COVID-19, where briefings and huddles supported experiential learning and team cohesion [35, 36]. Post-shift debriefings further promoted emotional processing, well-being, and retention by fostering resilience amid crisis-related fears; video-based formats sustained connections despite distancing [37].​​ Bidirectional communication as well as consolidation of information before dissemination and consistency of information were shown to be important factors to reduce stress and anxiety in ED staff [38]. Visual dashboards complemented these efforts by mitigating overload and improving guideline adherence. Arnold and colleagues were able to highlight in their review that visual dashboards can also positively influence the overload of information. Moreover, depending on the information shown, they can contribute to improving structural awareness and adherence to evidence-based safety guidelines [39].

Additional stressors were the fear of infection and the associated new role identity — from “helpers” to perceived “endangerers”— to patients and family, exacerbated by initial personal protective equipment shortages and sicker patients in wave 2. To address the perceived fears and to cope with the new challenges, some employees would have appreciated preventive offerings and psychological support. In the future, these offerings should be established as a fixed part of the work routine during crises and conducted within working hours. Simic et al. developed a holistic ED nursing support framework to provide better support of staff, build positive cultures and prepare ED managers in developing protocols that support the wellbeing of nurses during times of future crisis [12]. Regular pool-testing of SARS-CoV-2 and individualized work scheduling, that took into account the individual risk profiles of employees, have also proven to be helpful measures in reducing anxiety and stress among staff members. These pooled tests, where multiple employees submit their samples collectively for analysis on average three times a week ensured that employees were aware of their infection status. Employees felt safer both at work and in their private lives, as infections were detected early and their individual infection status was known. The fear of infecting colleagues or unknowingly carrying the infection into private life was positively influenced by the pool tests. Moreover, given the pool-testing design of testing staff members, they reduce the number of tests as well as financial expenditures and are quick to implement in case of pandemic events [40].

Pre-triage screening — resulting in 12-minute delays for positives — and dedicated dispatchers improved early infection detection while addressing crowding. Procedures need to be developed for identifying infectious patients is also a priority, as it reduces staff anxiety, provides certainty in how to act and improves patient flow. In the future, EDs should develop systems in which infectious patients can be reported before arriving at the ED. This provided staff with a little more predictability in the allocation of capacity and could better coordinate their personnel resources. Assistants for personal protective equipment delivery, floor markings for distancing, external testing, and ICU coordinators streamlined discharge and resource use. Once the patient is admitted to the ED the role of a dispatcher, who keeps an eye on the capacity of the isolation rooms and directs the flow of patients, was described as a special resource. In addition, the position of an assistant could be implemented during the pandemic. An assistant supports colleagues in patient care and provides additional materials to isolation rooms if needed. This also saves resources, as colleagues did not have to repeatedly put personal protective equipment on and off. The position of the assistant does not necessarily have to be filled by a nurse, depending on the assigned tasks, a skill mix (health science students, paramedics or physician assistance) is also possible [41]. This could enable EDs to reduce the already high pressure on staff during pandemics, support employees on-site, and conserve resources. EDs must also establish processes to reduce pandemic-related crowding. For patients with a test request who independently visit EDs, the establishment of an external testing site has proven to be effective [13]. This ensured that ED staff could focus their resources on more complex patient care and influence crowding in the ED positively; extending the test offer to employees could also reduce the workload of ED staff. Reducing the number of people in the ED by excluding relatives facilitates patient care through more continuous support for patients with fewer interruptions. Winters and colleagues were able to demonstrate high acceptance of this approach among ED staff: 61% of respondents were convinced that excluding relatives contributes to infection control [42]. However, clear rules must be established which also address ethical considerations; exceptions should be made for individuals nearing death [42] and other vulnerable groups like children and patients with dementia. For faster transfer of patients into inpatient care, the COVID-19 Intensive Care Coordinator proved helpful. It became possible to inquire about the joint capacities of all ICUs at Charité – Universitätsmedizin Berlin with just one phone call to the central coordination office, thus expediting the transfer of intensive care patients or directly transferring them from the ED to the ICU was facilitated. The ability to transfer hospitalized patients waiting for their test results to a ward for nonurgent cases, was perceived as a significant relief. After completing diagnostics, these patients were transferred from the ED to the exclusion ward until their final transfer to the target ward, which alleviated crowding.

An interdisciplinary approach that integrates communication, emotional support for employees, and operational processes to counteract crowding is essential for future emergency preparedness.

### Limitations

Our research was carried out in two urban EDs affiliated with tertiary care hospitals and the findings therefore represent the perceived realities of EDs in urban settings. It’s important to note that experiences in rural areas may differ significantly, necessitating further investigation for a more comprehensive understanding. We employed purposeful sampling, a method sometimes criticized for potentially overlooking certain aspects of the phenomenon under study due to participant selection. However, our study population exhibited considerable diversity in terms of age, sex and work experience in EDs. This heterogeneity suggests that our results offer a broad view of the perceived work realities in these settings. Conducting interviews in the ED setting can be viewed critically. Disruptions by colleagues, time pressure, or interruptions due to work obligations may negatively impact the openness of the interviewees. In contrast, however, this approach offers simpler practical implementation for both researchers and participants, as well as the advantage of an authentic and familiar environment. Moreover, interviews were conducted either before or after shifts in rooms specifically booked for the interviews, thereby minimizing any interruptions from duties or colleagues. The secondary data analysis has limitations that require careful consideration. First of all, the quality of the evaluated data set was influenced by the documentation quality and documentation routine of the ED staff. Due to the pandemic situation, it can be assumed that new documentation routines required a certain amount of time to adapt. Missing data occurred for age in 0.2% of visits, for referrals to the ICU in 0.1% of visits and for the urgency of treatment in 3.1% of visits. Despite the missing data, information on in- hospital mortality and COVID-19 status can be regarded as fully recorded. A large number of statistical tests were conducted across multiple outcomes, reflecting the exploratory nature of these analyses. While this approach facilitated comprehensive examination of the data, it increases the risk of Type I error due to multiple comparisons. However, this risk can be considered low in the present study, as multiple testing occurred only in isolated instances, while other statistically appropriate tests — which did not involve multiple comparisons — were predominantly used.

## Conclusions

The COVID‑19 pandemic had a profound impact on daily work in EDs. In addition to altered patterns of ED utilization, the pandemic particularly affected staff perceptions of workload and strain. Two overarching dilemmas characterized everyday work during the pandemic: (1) optimal care while conserving capacities and (2) optimal treatment while adhering to hygiene guidelines. Furthermore, several key areas were identified to strengthen future pandemic preparedness in EDs, with a focus on communication processes, staff safety, early detection measures, hygiene protocols, and resource management. The strategies highlight that effective crisis management requires an interdisciplinary approach: in addition to medical expertise, clear communication channels, evidence-based protective measures for staff and transparent resource allocation are crucial. Only through a systematic, multidimensional strategy can EDs be rendered resilient to future pandemics.

## Supplementary Information

Below is the link to the electronic supplementary material.

Supplementary Material 1

## Data Availability

The datasets generated and analyzed during the current study are not publicly available due to privacy or ethical concerns, but are available from the corresponding author on reasonable request.

## References

[1] Marani M, et al. Intensity and frequency of extreme novel epidemics. Proc Natl Acad Sci U S A. 2021;118(35). 10.1073/pnas.2105482118 PMC8536331 34426498

[2] Haileamlak A. Pandemics Will be More Frequent. Ethiop J Health Sci. 2022;32(2):228. 35693574 10.4314/ejhs.v32i2.1 PMC9175207

[3] WHO. The transition from the acute phase of COVID 19 working towards a paradigm shift for pandemic preparedness and response in the WHO European Region implementation report 2023 2024 Copenhagen. Copenhagen: WHO Regional Office for Europe; 2025.

[4] Mahajan P, et al. A Global Survey of Emergency Department Responses to the COVID-19 Pandemic. West J Emerg Med. 2021;22(5):1037–44. 34546878 10.5811/westjem.2021.3.50358 PMC8463065

[5] Köppen J, Hartl K, Maier CB. Health workforce response to Covid-19: What pandemic preparedness planning and action at the federal and state levels in Germany? Int J Health Plann Manag. 2021;36(S1):71–91. 10.1002/hpm.3146 PMC8250947 33735509

[6] Maccaro A, et al. Pandemic preparedness: a scoping review of best and worst practices from COVID-19. Healthc (Basel). 2023;11(18). 10.3390/healthcare11182572 PMC10530798 37761769

[7] Ramshorn-Zimmer Alexandra PM, Thomas H, Florian L, Markus Z. Brokmann Jörg Christian, Bernhard Michale, Gries André *Coronapandemie: Rolle der Zentralen Notaufnahme*. Dtsch Arztebl. 2020;117:A–1040.

[8] Holtz HK, et al. The Long Tail of COVID-19: Implications for the Future of Emergency Nursing. J Emerg Nurs. 2023;49(2):198–209. 36503829 10.1016/j.jen.2022.10.006 PMC9584853

[9] Jackson MR, et al. If I Can’t Do It, Who Will? Lived Experiences of Australian Emergency Nurses During the First Year of the COVID-19 Pandemic. J Emerg Nurs. 2023;49(5):733–43. 37294260 10.1016/j.jen.2023.05.004 PMC10186981

[10] Jerg-Bretzke L, et al. Psychosocial impact of the COVID-19 pandemic on healthcare workers and initial areas of action for intervention and prevention-the egePan/VOICE study. Int J Environ Res Public Health. 2021;18(19). 10.3390/ijerph181910531 PMC8508196 34639831

[11] Morawa E, et al. Psychosocial burden and working conditions during the COVID-19 pandemic in Germany: The VOICE survey among 3678 health care workers in hospitals. J Psychosom Res. 2021;144:110415. 33743398 10.1016/j.jpsychores.2021.110415 PMC7944879

[12] Simic MR, et al. ‘It’s only a matter of time’ - Lessons learnt and recommendations from COVID-19 to inform emergency nursing for future pandemics: an integrated literature review. Australas Emerg Care. 2024. 10.1016/j.auec.2024.10.004 39603854

[13] Augustin M, et al. Rapid response infrastructure for pandemic preparedness in a tertiary care hospital: lessons learned from the COVID-19 outbreak in Cologne, Germany, February to March 2020. Euro Surveill. 2020;25(21). 10.2807/1560-7917.ES.2020.25.21.2000531 PMC7268272 32489176

[14] Creswell JW, Plano Clark VL. Designing and conducting mixed methods research / John W. Creswell (Department of Family Medicine, University of Michigan), Vicki L. Plano Clark (School of Education, University of Cincinnati). Third edition, international student edition ed. 2018, Los Angeles: Sage.

[15] Tashakkori A, Teddlie C. SAGE Handbook of Mixed Methods in Social & Behavioral Research. California: Thousand Oaks; 2010.

[16] Greene JC, Caracelli VJ, Graham WF. Toward a Conceptual Framework for Mixed-Method Evaluation Designs. Educational Evaluation Policy Anal. 1989;11(3):255–74.

[17] Lall D. Mixed-methods research: Why, when and how to use. Indian J Continuing Nurs Educ. 2021;22(2):143–7.

[18] Palinkas LA, et al. Purposeful Sampling for Qualitative Data Collection and Analysis in Mixed Method Implementation Research. Adm Policy Ment Health. 2015;42(5):533–44. 24193818 10.1007/s10488-013-0528-y PMC4012002

[19] Helfferich C. Die Qualität qualitativer Daten. Manual für die Durchführung qualitativer Interviews. Vol. 4. Auflage 2011: VS Verlag für Sozialwissenschaften.

[20] Rädiker S. K.U., Analyse qualitativer Daten mit MAXQDA, Text, Audio und Video. Springer VS; 2019.

[21] Schilling J, et al. Retrospektive Phaseneinteilung der COVID-19-Pandemie in Deutschland bis Februar 2021. 2021(15): pp. 3–12.

[22] Braun V, Clarke V. Thematic analysis: a practical guide. SAGE; 2021.

[23] Braun V, Clarke V. Using thematic analysis in psychology. Qualitative Res Psychol. 2006;3(2):77–101.

[24] Vaismoradi M, Turunen H, Bondas T. Content analysis and thematic analysis: Implications for conducting a qualitative descriptive study. Nurs Health Sci. 2013;15(3):398–405. 23480423 10.1111/nhs.12048

[25] Quan H, et al. Updating and Validating the Charlson Comorbidity Index and Score for Risk Adjustment in Hospital Discharge Abstracts Using Data From 6 Countries. Am J Epidemiol. 2011;173:676–82. 21330339 10.1093/aje/kwq433

[26] Quan H, et al. Coding algorithms for defining comorbidities in ICD-9-CM and ICD-10 administrative data. Med Care. 2005;43(11):1130–9. 16224307 10.1097/01.mlr.0000182534.19832.83

[27] Charlson M, et al. Validation of a combined comorbidity index. J Clin Epidemiol. 1994;47(11):1245–51. 7722560 10.1016/0895-4356(94)90129-5

[28] Charlson ME, et al. A new method of classifying prognostic comorbidity in longitudinal studies: Development and validation. J Chronic Dis. 1987;40(5):373–83. 3558716 10.1016/0021-9681(87)90171-8

[29] Slagman A, et al. Identification of low-acuity attendances in routine clinical information documented in German Emergency Departments. BMC Emerg Med. 2023;23(1):64. 37280527 10.1186/s12873-023-00838-2 PMC10243890

[30] Schranz M, et al. Changes in emergency department utilisation in Germany before and during different phases of the COVID-19 pandemic, using data from a national surveillance system up to June 2021. BMC Public Health. 2023;23(1):799. 37131165 10.1186/s12889-023-15375-7 PMC10152015

[31] Bergrath S, et al. Impact of the COVID-19 pandemic on emergency medical resources: an observational multicenter study including all hospitals in a major urban center of the Rhein-Ruhr metropolitan region. Anaesthesist. 2021: pp. 1–9. 10.1007/s00101-021-01005-7 PMC8296826 34292358

[32] Suárez V, et al. [University emergency departments in the corona pandemic-Results from the ReCovER registry]. Med Klin Intensivmed Notfmed, 2021: pp. 1–8. 10.1007/s00063-021-00859-4 PMC8408818 34468772

[33] Oh D-Y, et al. SARS-CoV-2-Varianten: Evolution im Zeitraffer. Dtsch Arztebl Int. 2021;118(9):460.

[34] Eckert N, Lenzen-Schulte M. Vorerkrankungen: Risikogruppen sind jetzt bekannt. Dtsch Arztebl Int. 2020;117(43):A2047–8.

[35] Stafford JL, et al. Clinical debriefing during the COVID-19 pandemic: hurdles and opportunities for healthcare teams. Adv Simul (Lond). 2021;6(1):32. 34526150 10.1186/s41077-021-00182-0 PMC8441031

[36] Paquay M, et al. A success story of clinical debriefings: lessons learned to promote impact and sustainability. Front Public Health. 2023;11:1188594. 37475771 10.3389/fpubh.2023.1188594 PMC10354544

[37] Monette DL, et al. A Video-based Debriefing Program to Support Emergency Medicine Clinician Well-being During the COVID-19 Pandemic. West J Emerg Med. 2020;21(6):88–92. 33052815 10.5811/westjem.2020.8.48579 PMC7673898

[38] Sangal RB, et al. Leadership communication, stress, and burnout among frontline emergency department staff amid the COVID-19 pandemic: A mixed methods approach. Healthcare. 2021;9(4):100577. 34411923 10.1016/j.hjdsi.2021.100577 PMC8361146

[39] Arnold M, Goldschmitt M, Rigotti T. Dealing with information overload: a comprehensive review. Front Psychol. 2023;14:1122200. 37416535 10.3389/fpsyg.2023.1122200 PMC10322198

[40] Schulte PA, et al. Considerations for Pooled Testing of Employees for SARS-CoV-2. J Occup Environ Med. 2021;63(1):1–9. 33378322 10.1097/JOM.0000000000002049 PMC7773162

[41] Pourmand A, et al. Rethinking Traditional Emergency Department Care Models in a Post-Coronavirus Disease-2019 World. J Emerg Nurs. 2023;49(4):520–e5292. 37086252 10.1016/j.jen.2023.02.008 PMC10116161

[42] Winters RB, et al. The Impact of Coronavirus Disease 2019 Visitor Restrictions on the Attitudes of Emergency Department Staff. J Emerg Nurs. 2024;50(1):106–16. 37452812 10.1016/j.jen.2023.06.010

