# Supplementary material for: Investigating changes in care patterns and lessons learned during COVID-19 pandemic – an exploratory, convergent mixed method study at two university emergency departments in Germany
Source: BMC Emerg Med. 2026 Mar 10;26:98. doi: 10.1186/s12873-026-01528-5 (PMC13064274; doi:10.1186/s12873-026-01528-5)
Supplement: Supplementary file 1 — Supplementary Material 1 [file 12873_2026_1528_MOESM1_ESM.pdf]

Table_S1: Estimates from the fixed-effects model for the distribution of the weekly utilization of the ED (% of weekly ED-visits on the specific weekday)

Parameter	Estimate (95%-CI*)	p-value
Intercept	13.90 (12.01;15.79)	<0.001
Weekday (ref=Monday)		
Tuesday	-0.13 (-0.77;0.51)	0.691
Wednesday	-0.54 (-1.18;0.10)	0.096
Thursday	-0.64 (-1.28;-0.01)	0.048
Friday	0.54 (-0.10;1.18)	0.095
Saturday	2.76 (2.13;3.40)	<0.001
Sunday	1.12 (0.49;1.76)	0.001
Wave (ref=2019)		
1st COVID wave	-0.29 (-1.19;0.60)	0.523
2nd COVID wave	1.01 (0.29;1.72)	0.006
Interactions (ref= respective weekday*2019)		
Tuesday * 1st COVID-19 wave	-0.12 (-1.39;1.14)	0.847
Tuesday * 2nd COVID-19 wave	-1.20 (-2.20;-0.20)	0.019
Wednesday * 1st COVID-19 wave	0.35 (-0.91;1.62)	0.584
Wednesday * 2nd COVID-19 wave	-0.54 (-1.55;0.46)	0.286
Thursday * 1st COVID-19 wave	0.88 (-0.38;2.15)	0.172
Thursday * 2nd COVID-19 wave	-0.86 (-1.87;0.14)	0.090
Friday * 1st COVID-19 wave	1.56 (0.29;2.82)	0.016
Friday * 2nd COVID-19 wave	-0.64 (-1.64;0.36)	0.211
Saturday * 1st COVID-19 wave	-0.57 (-1.84;0.69)	0.374
Saturday * 2nd COVID-19 wave	-2.34 (-3.35;-1.34)	<0.001
Sunday * 1st COVID-19 wave	-0.47 (-1.74;0.79)	0.462
Sunday * 2nd COVID-19 wave	-1.86 (-2.86;-0.86)	<0.001

*Confidence interval
